# Rational design of a water-soluble NIR AIEgen, and its application in ultrafast wash-free cellular imaging and photodynamic cancer cell ablation†
†Electronic supplementary information (ESI) available: Compound characterization, PL spectra of TTVP in solvents with different polarities, plasma membrane-imaging of other cells, and morphological changes of the plasma membrane upon light irradiation. See DOI: 10.1039/c7sc04963c


**DOI:** 10.1039/c7sc04963c

**Published:** 2018-03-13

**Authors:** Dong Wang, Huifang Su, Ryan T. K. Kwok, Xianglong Hu, Hang Zou, Qianxin Luo, Michelle M. S. Lee, Wenhan Xu, Jacky W. Y. Lam, Ben Zhong Tang

**Affiliations:** a Hong Kong Branch of Chinese National Engineering Research Center for Tissue Restoration and Reconstruction , Department of Chemistry , Institute of Molecular Functional Materials , State Key Laboratory of Neuroscience , Division of Biomedical Engineering , Division of Life Science , The Hong Kong University of Science and Technology , Clear Water Bay , Kowloon , Hong Kong , China . Email: tangbenz@ust.hk; b MOE Key Laboratory of Laser Life Science & Institute of Laser Life Science , College of Biophotonics , South China Normal University , Guangzhou , 510631 , China . Email: xlhu@ust.hk ; Email: xlhu@scnu.edu.cn; c Department of Osteology , The First Affiliated Hospital of Zhengzhou University , Zhengzhou University , Zhengzhou 450000 , PR China; d Center for AIE Research , College of Materials Science and Engineering , Shenzhen University , Shenzhen 518060 , China

## Abstract

The synthesis of water-soluble near-infrared (NIR)-emissive fluorescent molecules with aggregation-induced emission (AIE) characteristics and theranostic functions is highly desirable but remains challenging. In this work, we designed and readily prepared for the first time such a molecule with AIE features, good water-solubility and intense emission in the NIR region. This AIE luminogen (AIEgen) is able to specifically “light up” the cell membrane without the involvement of a washing procedure. Interestingly, the staining process can be performed by simply shaking the culture with cells at room temperature for only a few seconds after the addition of the AIEgen, indicating an ultrafast and easy-to-operate staining protocol. This is the first fluorescent “light-up” probe for cell-imaging that allows the combination of a short staining period (at the second-level) with a wash-free process. Additionally, the presented AIEgen has also been developed to serve as an excellent phototherapeutic agent for high efficiency generation of reactive oxygen species (ROS) upon visible light irradiation, which allows its effective application in the photodynamic ablation of cancer cells, demonstrating its dual role as an imaging and phototherapeutic agent.

## Abstract 2

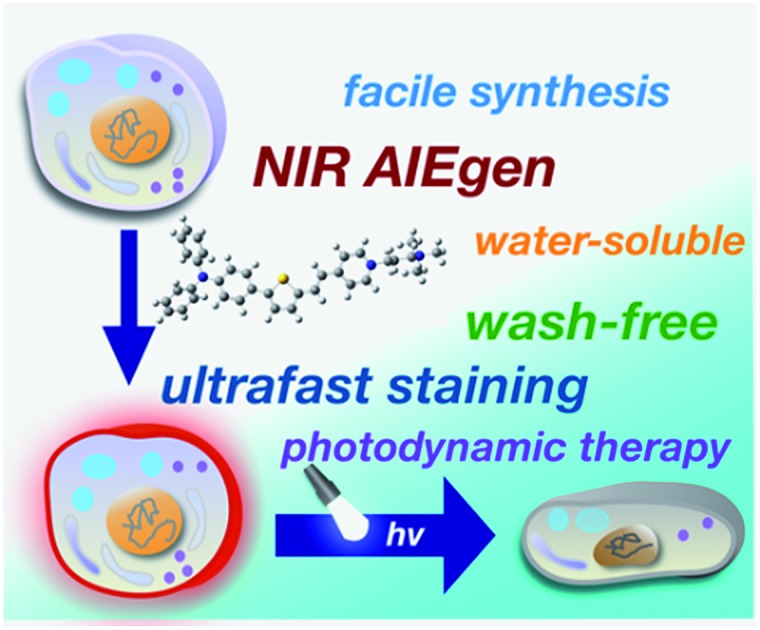
The first water-soluble NIR AIEgen was synthesized and used for ultrafast wash-free cellular imaging and photodynamic cancer cell ablation.

## Introduction

Fluorescence bioimaging techniques have emerged as powerful and non-invasive analytical tools for visualizing biological species by virtue of their fast response, excellent temporal resolution, superb sensitivity, *in situ* workability, simple operation, and good reproducibility.^1^–^3^ As a major branch of fluorescent materials, small organic fluorophores are nowadays undergoing an explosive development, particularly fluorophores with NIR emission (>700 nm), which possess distinct advantages such as high penetration depth, low biological autofluorescence interference, minimal photodamage to biological structures, and reduced light scattering.^4^–^9^ However, owing to the π–π stacking and other non-radiative pathways, conventional NIR fluorophores are either weakly emissive or non-emissive at high concentration or in the aggregation state. This phenomenon, known as aggregation-caused quenching (ACQ),^10^–^12^ is quite common and remains the major barrier to their practical applications in the fields of bioimaging and theranostics, since organic molecules naturally aggregate in biological media owing to the high hydrophobicity of their emitting centers. Interestingly, the emergence of a novel class of NIR fluorophores with aggregation-induced emission (AIE) characteristics perfectly solves the ACQ problem.^13^ AIE luminogens (AIEgens) are non-emissive when molecularly dissolved in solvents, but exhibit intense fluorescence in the aggregation state.^14^,^15^ The AIE features permit the use of fluorophore solution at any concentrations, and enable the development of fluorescent “light-up” probes for biosensing and imaging applications.

The current development of NIR AIEgens is far from ideal, and until now, only a handful of AIEgens exhibiting high-performance NIR emission have been developed and used in biological studies.^16^–^18^ Furthermore, considering that biological research studies are conducted in physiological environments or aqueous media, the utilization of water-soluble AIEgens holds an intrinsic advantage. Although some water-soluble AIEgens with short-wavelength emissions have been prepared and employed as powerful bioprobes,^19^–^23^ to the best of our knowledge, there have been no previous reports on water-soluble NIR AIEgens. Developing NIR AIEgens with good water-solubility remains an important and challenging task, even though considerable efforts have been devoted by the scientific community.

*In vitro* cellular imaging, that is one of the most widely used applications of fluorescence bioimaging techniques, has become indispensable for biological analysis and clinical diagnosis. As an important cell organelle, the plasma membrane that consists of the phospholipid bilayer is a protective two-dimensional boundary between a living cell and its surroundings. The plasma membrane has been demonstrated to be involved in various cellular processes and bio-functions, such as cell signaling, cell adhesion, endocytosis, exocytosis and selective permeation of substances.^24^,^25^ The abnormality of the cell plasma membrane is a critical biomarker for cell status and many diseases. Therefore, visualizing the plasma membrane using fluorescent bioprobes must be significantly important and useful. However, previously developed plasma membrane-specific fluorophores (such as DiO, DiI, and CellMask) have their respective and collective drawbacks including short emission wavelengths, small Stokes shifts, requirement of hazardous organic solvents for preparing stock solution, long incubation periods and tedious washing procedures after cell staining.^26^–^29^ In particular, the latter two shortcomings have been long-term unresolved issues in cellular fluorescence imaging. Long incubation is time-consuming, and often causes nonspecific illumination of cellular components.^30^ Aiming to improve the signal-to-noise (S/N) ratio of cell imaging, a washing process after cell staining is usually required for eliminating the strong residual signal from the free dyes. The post-washing process could result in some problems, for instance, delaying the acquisition of microscopic data, decreasing the accuracy of cell-imaging results due to both the altered cellular environment and the loss of cells. Moreover, the washing procedure is incompatible with continuous sensing or monitoring of biological processes.^31^,^32^ Additionally, the plasma membrane is considered to be a wonderful cellular targeting site for implementing therapeutic applications^33^ because the plasma membrane is strongly related to various cellular processes, and is the outermost protection layer of cells, due to which its destruction is fatal to cells. However, almost all of the previously reported plasma membrane-staining fluorophores can only be used as imaging probes instead of dual applications in simultaneous imaging and therapy. Evidently, developing a novel fluorescent plasma membrane probe that overcomes the above-mentioned deficiencies would be remarkably important and urgently needed.

In this contribution, we report for the first time the design and facile synthesis of a water-soluble AIEgen (named TTVP) with emission in the NIR region. The plasma membrane can be specifically targeted by TTVP through a wash-free and ultrafast staining procedure, and furthermore, image-guided photodynamic cancer cell ablation was successfully achieved upon visible light irradiation.

## Results and discussion

### Design and synthesis

**Scheme 1:**
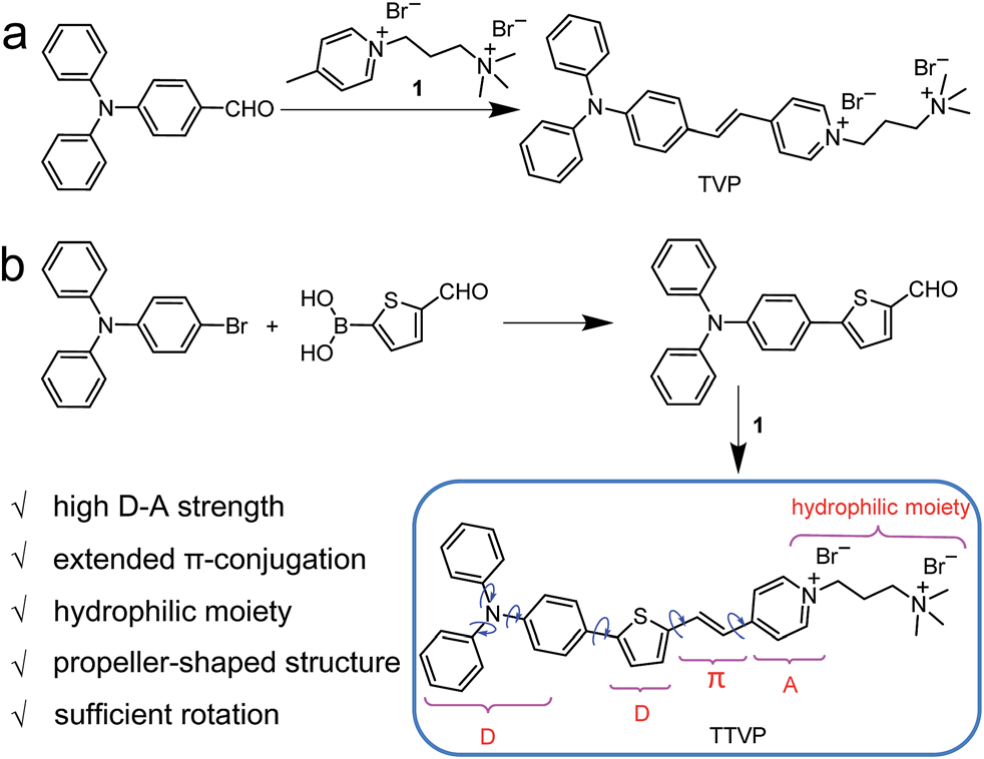
(a) Synthetic route to TVP. (b) Design rationale and synthetic route to water-soluble NIR AIEgen TTVP.

Common synthetic protocols of fluorophores with long emission wavelengths include the connection of electron-accepting (A) units with electron-donating (D) units *via* π-bridge(s), the extension of π-conjugation, and the incorporation of these two strategies. In this work, the designed molecules, TVP and TTVP, are both D–A type compounds comprising a triphenylamine segment (working as D) or/and a thiophene fragment (D and π-bridge), a carbon–carbon double bond (π-bridge), and pyridinium (A). The ingenious combination of a strong electron donor–acceptor (D–A) interaction with extended π-conjugation in these structures could facilitate intramolecular charge transfer (ICT), therefore resulting in lower electronic bandgaps, as well as longer absorption and emission wavelengths. The preparation of water-soluble NIR AIE luminogens could be achieved by combining high donor–acceptor (D–A) strength and hydrophilic units into a propeller-shaped molecular structure (Scheme 1).

As depicted in Scheme 1, TVP was facilely synthesized by a one-step reaction of 4-(diphenylamino)benzaldehyde with 4-methyl-1-(3-(trimethylammonio)propyl)pyridin-1-ium bromide, with a yield of 72%. TTVP was produced by a two-step reaction. The Suzuki–Miyaura coupling reaction of 4-bromo-*N*,*N*-diphenylaniline with (5-formylthiophen-2-yl)boronic acid smoothly proceeded and generated 5-(4-(diphenylamino)phenyl)thiophene-2-carbaldehyde, which then underwent a condensation reaction with the pyridinium salt, facilely giving TTVP with a total yield of 65%.

### Photophysical properties

**Fig. 1:**
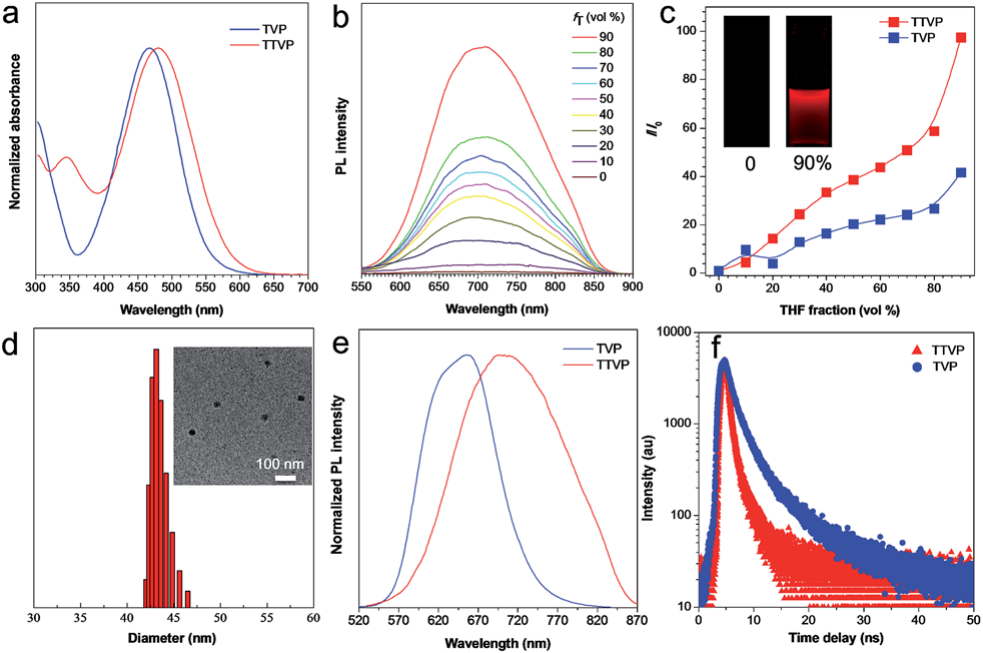
(a) Normalized absorption spectra of TVP and TTVP in aqueous solutions. (b) PL spectra of TTVP in water/THF mixtures with different THF fractions (*f*_T_). Concentration: 10 μM; excitation wavelength: 480 nm. (c) The plot of the emission maximum and the relative emission intensity (*I*/*I*_0_) *versus* the composition of the water/THF mixtures of TVP and TTVP. Inset: fluorescence photographs of TTVP in the aqueous solution and in water/THF mixtures with 90% THF fractions under 365 nm UV irradiation. (d) Particle size distributions of TTVP aggregates in the water/THF mixture with a 90% THF fraction. Concentration: 10 μM. Inset: TEM spectrum of TTVP aggregates in the water/THF mixture with a 90% THF fraction. (e) Normalized PL spectra of TVP and TTVP in the solid state. (f) Fluorescence decay curves of TVP and TTVP in the solid state.

**Fig. 2:**
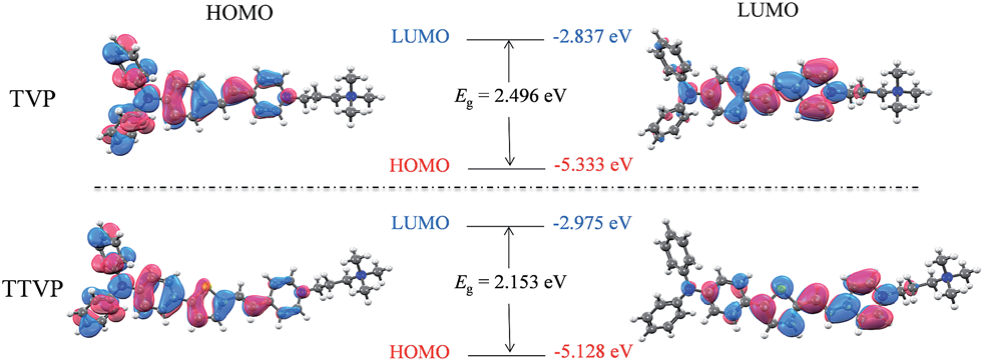
Molecular orbital amplitude plots of the HOMO and LUMO energy levels of TVP and TTVP.

Both TVP and TTVP have good water solubility benefiting from the existence of both positively charged quaternary ammonium and pyridinium having hydrophilic characteristics, as well as the small size of the hydrophobic moiety. The aqueous solutions of TVP and TTVP display maximum absorption bands at 467 nm (with a 25 070 M^–1^ cm^–1^*molar extinction coefficient*) and 480 nm (with a 33 517 M^–1^ cm^–1^*molar extinction coefficient*), respectively (Fig. 1a and Table S1†). The longer absorption wavelength of TTVP is ascribed to its smaller HOMO–LOMO energy gap than TVP (Fig. 2) due to the stronger electron donating–accepting interaction of the emitting center of TTVP, resulting from the existence of the thiophene fragment with electron-donating properties. Density functional theory (DFT) calculations also indicate that the electron density in the highest occupied molecular orbital (HOMO) is delocalized at the triphenylamine segment, while the pyridinium unit dominates the lowest unoccupied molecular orbital (LUMO).

The investigation of AIE features in water/THF mixtures with different THF fractions (*f*_T_) demonstrates that both TVP and TTVP are typical AIE-active molecules. They are almost non-emissive in aqueous solutions in the single-molecule state, mainly because the rotational motions of molecular rotors (such as the phenyl rings of the triphenylamine moiety, carbon–carbon double bonds, and pyridinium and thiophene units) consume exciton energy and increase nonradiative decay rates, leading to non-emission. And the photoluminescence (PL) intensities gradually increase with increasing the fraction of THF due to the formation of nanoaggregates (Fig. 1b). The strongest PL intensities were observed at a 90% fraction of THF upon aggregation, in which the PL intensities were enhanced to about 41.7- and 97.3-fold compared with that of aqueous solutions (Fig. 1c). The significantly enhanced emissions in aggregates could be attributed to the fact that the restriction of the rotor motions activates radiative decay; meanwhile, the twisted conformation of the triphenylamine segment can extend the intermolecular distance and prevent emission quenching by the reduction of the intermolecular π–π interaction, thus switching on the luminescence process in the aggregation state. Their maximum emissions in the aggregation state are located at 629 and 708 nm, respectively, with 4.4% and 1.7% quantum yields, indicating both their red/NIR-emission properties and large Stokes shifts. Moreover, dynamic light scattering (DLS) analysis and/or transmission electron microscopy (TEM) measurements were performed to confirm the formation of aggregates upon the addition of THF into the aqueous solutions. DLS reveals that the average hydrodynamic diameters of these nanoaggregates that formed in the suspension containing a 90% fraction of THF are around 46 for TVP, and 43 nm for TTVP (Fig. S8† and 1d), while their spherical morphology was observed by TEM analysis. In the solid state, the PL spectra of TVP and TTVP peaked at 657 and 705 nm, respectively (Fig. 1e), while their fluorescence decay curves reveal that their lifetimes are 5.75 and 0.92 ns (Fig. 1f). In addition, the solvatochromism study shows that with the increase of solvent polarity, the emission maximum of TTVP largely red shifts while the emission intensity was considerably reduced (Fig. S9†), suggesting a strong twisted intramolecular charge transfer (TICT) effect.^34^

### Bio-imaging and therapy

**Fig. 3:**
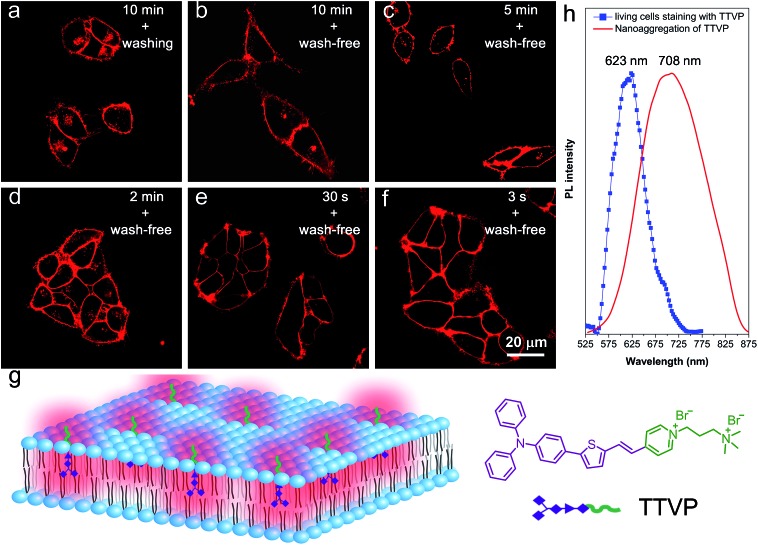
Confocal images of living HeLa cells after incubation with TTVP (500 nM) for (a) 10 min by the use of a washing procedure after incubation, (b) 10 min, (c) 5 min, (d) 2 min, (e) 30 s, and (f) an extremely short incubation period (around 3 s) by the use of the wash-free procedure after incubation. *λ*_ex_: 488 nm (1% laser power). (g) Schematic illustration of plasma membrane-specific imaging with TTVP. Scale bar = 20 μm. (h) Fluorescence spectrum of the plasma membrane of living HeLa cells stained with TTVP, and the fluorescence spectrum of TTVP nanoaggregation in the THF/H_2_O mixture.

As a water-soluble NIR-emissive AIEgen, TTVP maintains an “off” state in an aqueous environment and thus has great potential to serve as a “light-up” probe for bioimaging with minimal background interference from both free dyes and *bio*-substrate autofluorescence. In this preliminary bioimaging experiment, the cell imaging study was proceeded by using HeLa cells as a cell model, and incubating 500 nM of TTVP for 10 min. It was observed that the plasma membrane can be clearly visualized with excellent image contrast to the cell background regardless of the washing or non-washing process after cell staining (Fig. 3a and b). The influence of the incubation period was then investigated by utilizing the wash-free procedure with different staining times. The results demonstrated that no obvious change of fluorescence imaging quality in terms of both fluorescence intensity and specificity was found with the reduction of staining time from 10 min to 30 s (Fig. 3b–e). Surprisingly, the plasma membrane was strongly lit up *via* the facile staining process of simply shaking the cell culture with TTVP for a few seconds at room temperature (Fig. 3f), indicating its ultrafast staining (at the second-level) characteristic. On the other hand, when the staining time was increased to 4 h, the plasma membrane can still be clearly visualized; in addition, a majority of TTVP can enter the cells after staining for 6 h, and the cell internal showed strong emission (Fig. S10†). It is believed that the plasma membrane-staining ability of TTVP could be mainly attributed to both its positively charged characteristic and amphiphilic properties. The positive charges of TTVP enable it to bind with cells through electrostatic interactions, especially with cancer cells that generally possess a negatively charged surface.^35^ TTVP is not able to pass through the hydrophobic region of phospholipid bilayers within a short period due to its good hydrophilicity. Meanwhile, the hydrophobic emitting moiety of TTVP is embedded into the hydrophobic region with low polarity, evidently giving fluorescence emission upon irradiation according to the restriction of intramolecular motion (RIM) mechanism of the AIE process (Fig. 3g). Indeed, the maximum emission of living HeLa cells stained with TTVP is located at 623 nm (Fig. 3h); the blue-shifted emission from 708 to 623 nm should be caused by the low polarity of the surrounding environment of TTVP in living cells, strongly validating the hypothesis on the embedding of TTVP into the hydrophobic region of the plasma membrane. Additionally, ultrafast staining could result from the excellent monodispersity of TTVP in the culture media of cells.

**Fig. 4:**
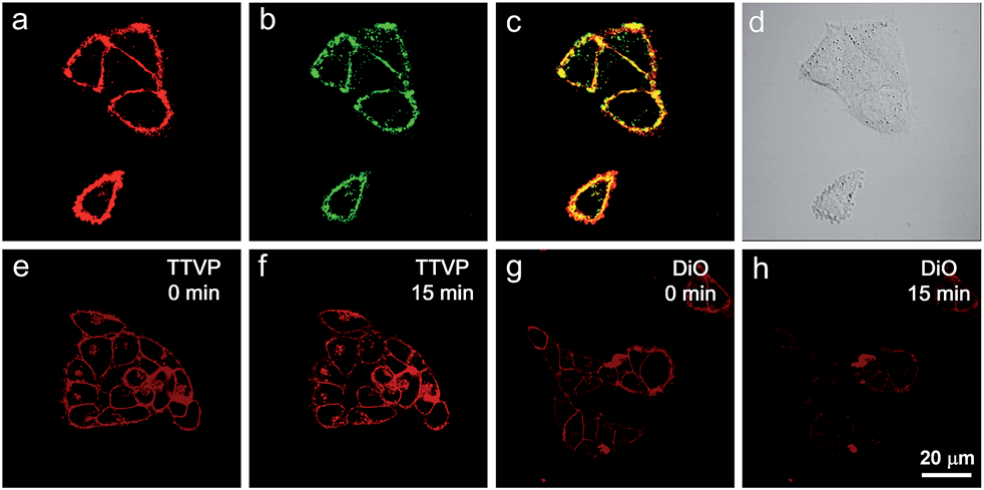
Co-localization imaging of HeLa cells stained with TTVP and DiO, and their photostabilities. Confocal images of HeLa cells stained with (a) TTVP and (b) DiO, and (c) merged images of panels (a) and (b), and (d) bright-field. Confocal images of HeLa cells (e and g) before (0 min) and (f and h) after laser irradiation for 15 min; confocal images of HeLa cells stained with (e and f) TTVP and (g and h) DiO. *λ*_ex_: 488 nm (1% laser power). Scale bar = 20 μm. The emission filter of TTVP: 600–744 nm; the emission filter of DiO: 490–600 nm.

The specificity of TTVP to the plasma membrane was evaluated by co-staining with DiO, which is a commercially available bioprobe for the plasma membrane. In this co-localization experiment, after incubating HeLa cells with DiO for 10 min, TTVP was added into the culture followed by culture shaking for a few seconds at room temperature. In order to accommodate the staining protocol of DiO, postwashing after cell staining was carried out. As shown in Fig. 4, TTVP can selectively accumulate at the plasma membrane and emit strong red fluorescence. The well-merged image between TTVP and DiO indicates good specificity for the plasma membrane, and the Pearson correlation coefficient is determined to be 89%. It was observed that TVP can also specifically stain the plasma membrane within a short incubation period (Fig. S11†). Moreover, aiming to assess the photostability of TTVP and DiO parallelly, continuous excitation and sequential scanning with a confocal microscope were performed. The result shows that the emission intensity of TTVP slightly decreased within 15 min irradiation (Fig. 4e and f), and the fluorescence loss of DiO is very obvious upon irradiation under the same conditions (Fig. 4g and h), demonstrating the superior photostability of TTVP to that of DiO. Furthermore, encouraged by the distinct advantages of TTVP for membrane-specific imaging, this ultrafast staining and wash-free cellular imaging protocol was further employed for staining other cell lines, including 293T, HCC827, HCT116, and MDCK2. In all tested cases, the plasma membrane was clearly visualized with a high S/N ratio of cell imaging with intense red emission (Fig. S12†), suggesting the good tolerance of TTVP to cell types.

**Fig. 5:**
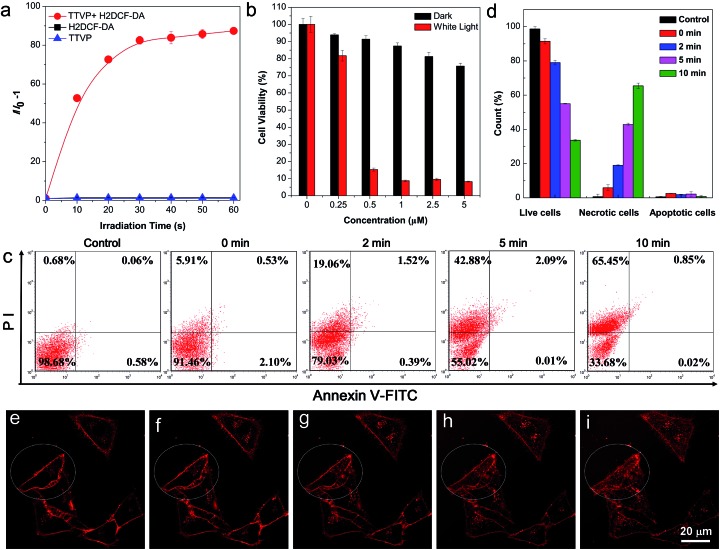
ROS generation upon white light irradiation and the PDT study of TTVP. (a) Relative change in fluorescence intensity (*I*/*I*_0_ – 1) at 534 nm of H2DCF-DA, TTVP, mixtures of H2DCF-DA and TTVP in PBS upon white light irradiation for different times. Concentrations: 10 μM (TTVP) and 5 μM (H2DCF-DA). Light power: 10 mW cm^–2^ (b) Cell viability of HeLa cells stained with different concentrations of TTVP in the absence or presence of white light irradiation for 10 min. Light power: 10 mW cm^–2^. (c) Cell apoptosis and necrosis analyzed using a flow cytometer with Annexin V-FITC/PI double staining after different treatments. Concentrations: 500 nM (TTVP). (d) Statistical analysis of flow cytometry data in (c). (e–i) Confocal images of living HeLa cells stained with TTVP through continuous laser irradiation. *λ*_ex_: 488 nm (20% laser power).

The strong absorption of TTVP in the visible light region could lead to the utilization of visible light as the excitation light source for photodynamic therapy (PDT) application, which is an appropriate and gentle approach for cancer therapy, and has been clinically approved for eliminating malignant tumor cells with minimal invasion and precise controllability.^36^–^39^ Visible light causes less damage to the biological system than UV light. The ROS generation efficiency of TTVP was initially determined by the use of H2DCF-DA as an indicator, which emits fluorescence with a “turn on” process triggered by ROS. As depicted in Fig. 5a, TTVP or H2DCF-DA alone was non-emissive or weakly emissive, and each fluorescence intensity remains almost constant during 60 s white light irradiation. In contrast, in the presence of TTVP, the emission intensity of H2DCF-DA gradually enhanced with increasing exposure time to white light, reaching 87-fold within 60 s. In addition, a high ^1^O_2_ quantum yield (80.16%) for TTVP was determined using a commercial ^1^O_2_ probe, 9,10-anthracenediyl-bis(methylene) dimalonic acid (ABDA), as the indicator, and employing Rose Bengal (RB) as the standard photosensitizer (Fig. S13†). The efficient ROS generation of TTVP could be attributed to both its small singlet–triplet energy gap (0.47 eV) and excellent monodispersity. The former favors the yield improvement of the triplet excited state, and the latter can enlarge the contact area between TTVP and oxygen. Moreover, it was observed that the ROS generation efficiency of TTVP was enhanced upon aggregate formation (Fig. S14†). The enhancement could be attributed to the increased intersystem crossing (ISC) rate and the improved yield of the triplet excitons, which result from the smaller singlet–triplet energy gap in the aggregation state.^39^,^40^ Furthermore, the ^1^O_2_ quantum yield (49.22%) of TVP is lower than that of TTVP due to the larger singlet–triplet energy gap (0.56 eV) of TVP. The effective ROS generation endows TTVP with prominent potential for PDT application, which was quantitatively evaluated on HeLa cells by a standard MTT assay. A dose-dependent toxicity was determined in both the absence and presence of white light irradiation. The results demonstrate that TTVP exhibits low cytotoxicity under dark conditions, which is one of the essential features of photosensitizers for PDT application. The HeLa cell viability dropped rapidly to 15% with a concentration of 500 nM, and 1 μM TTVP causes almost complete cell death under white light irradiation (Fig. 5b), indicating its remarkable efficiency for cancer cell ablation in the PDT pathway. In comparison, HeLa cells maintained 90% viability when they were incubated with 1 μM of TTVP under dark conditions. Furthermore, flow cytometric analysis using Annexin V-FITC/propidium iodide (PI) double staining was utilized to determine cell apoptosis (Fig. 5c and d).^41^ It was observed that light irradiation caused cancer cell necrosis in a short time, and the ratio of necrotic cells significantly increased with prolonging the irradiation time, which suggested the high efficiency of TTVP in photodynamic ablation of cancer cells. It is worth noting that continuous light irradiation with strong power (around 18.5-fold higher than that for cell imaging depicted in Fig. 3) led to some changes of cells, for instance, TTVP can gradually enter the cells (Fig. 5e–i); the cell membrane morphology changed, and the formation of blebs on the plasma membrane was clearly observed (Fig. S15†), which is a sign of cell death.^42^ These changes can be attributed to the fact that the ROS generated from TTVP considerably disrupt the rigidity and permeability of the plasma membrane, and induce cancer cell death. In addition, COS-7 normal cells were also employed for both cellular imaging and PDT application. It was found that TTVP can stain normal cells, but a higher concentration (5 μM) is needed for achieving clear cellular imaging (Fig. S16†). In comparison, when HeLa cancer cells were tested for cellular imaging, 0.5 μM of TTVP is able to provide very good quality of cellular imaging (see Fig. 3). The difference is perhaps caused by the more negatively charged surface of cancer cells.^35^ The positive charges of TTVP enable it to rapidly and efficiently bind with cancer cells through electrostatic interactions. The results of the PDT study showed that COS-7 normal cells maintained 33% cell viability when 20 μM of TTVP was used (Fig. S17†). In comparison, 0.5 μM of TTVP almost completely killed HeLa cancer cells. The big difference could be attributed to the much less efficient staining of TTVP with normal cells.

**Fig. 6:**
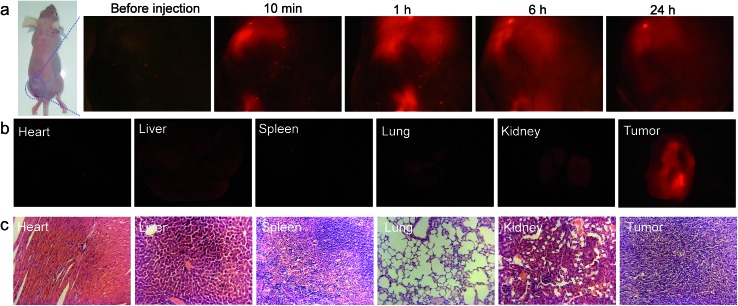
*In vivo* imaging and cytotoxicity test. (a) Biodistribution of TTVP in HeLa tumor-bearing mice after intratumoral injection of TTVP (10 μM, 20 μL) at different times. (b) *Ex vivo* fluorescence imaging of various organs and tumor tissue from mice injected with TTVP. The mice were sacrificed at 24 h post-injection. (c) Images of various H&E-stained organ slices from mice.

The *in vivo* imaging experiment and cytotoxicity test have also been carried out. As depicted in Fig. 6, TTVP clearly provided tumor imaging after intratumoral injection of TTVP aqueous solution in HeLa tumor-bearing mice. In order to evaluate the tumor retention potential of TTVP, the observation of tumor imaging for the duration from 10 min to 24 h after injection was performed. It was observed that the tumor site was continuously imaged upon intratumoral injection with TTVP. At 24 h post-injection, the tumor fluorescence was still significant for observation, suggesting the outstanding tumor retention properties of TTVP, possibly benefiting from the persistence of membrane insertion. In addition, TTVP can accumulate at tumors with good specificity at 24 h post-injection (Fig. 6b), and the images of H&E-stained organ slices showed that there were no obvious pathological changes of the main organs in TTVP treated mice, indicating its undetectable systemic toxicity.

## Conclusions

To sum up, we report for the first time the synthesis of a water-soluble NIR-emissive AIEgen (TTVP), which has long been a key limitation of AIE studies. Remarkably, TTVP can specifically and consistently stain the plasma membrane with excellent image contrast to the cell background by utilizing a wash-free procedure after an ultrafast staining process, which was performed by simply shaking the culture at room temperature for a few seconds after adding TTVP. The capability of the ultrafast staining of the plasma membrane could result from the fact that the water-monodispersed TTVP bearing positive charges can quickly diffuse and be embedded into the plasma membrane, and evidently “light-up” the plasma membrane according to the RIM mechanism of the AIE process. Additionally, both the water solubility and AIE characteristics synergistically enable the wash-free imaging protocol. Its extraordinary features for cell imaging, including NIR-emission, large Stokes shift, good photostability, ultrafast staining and a wash-free procedure, make this AIEgen far superior to commercially available dyes for plasma membrane-specific imaging. To the best of our knowledge, this is the first fluorescence “light-up” probe for cell-imaging allowing both ultrafast staining (a few seconds) and wash-free operations. Apart from the application of cell imaging, TTVP is also proven to be a powerful photosensitizer in ROS generation, enabling its exploration in cancer cell ablation through the PDT process. It was demonstrated that low concentrations of TTVP even down to 1 μM almost completely kill cancer cells upon white light irradiation, indicating its high efficiency for PDT application. Light controlled cancer cell killing combined with fluorescence emission in the NIR region enables TTVP to be an attractive candidate for imaging-guided PDT. In addition, TTVP can also be used for *in vivo* imaging thanks to its long wavelengths of both absorption and emission. The success for *in vivo* imaging will stimulate the development of water-soluble AIEgens with longer emission wavelengths for tumor therapy.

This study provides a new insight into the design of water-soluble AIEgens with long emission wavelengths for the development of efficient and easy-to-operate fluorescent bioprobes with “light up” nature. Our findings would also promote new strategies for the construction of efficient phototherapeutic molecules for the ablation of cancer cells, and facilitate the exploration of bioprobes for theranostic applications.

## Experimental procedures

### Materials and methods

Dulbecco's Modified Essential Medium (DMEM) and RPMI-1640 were purchased from Gibco (Life Technologies). Phosphate buffered saline (PBS), fetal bovine serum (FBS), penicillin, streptomycin, and DiO were purchased from Thermo Fisher Scientific. H2DCF-DA was purchased from Sigma-Aldrich. Pd(dppf)Cl_2_, piperidine, 4-bromo-*N*,*N*-diphenylaniline, (5-formylthiophen-2-yl)boronic acid, 3-bromo-*N*,*N*,*N*-trimethylpropan-1-aminium bromide, 4-(diphenylamino)benzaldehyde and 4-methylpyridine were purchased from Sigma-Aldrich, J&K and MERYER. All the chemicals were used as received without further purification. 1-(3-Trimethylammoniopropyl)-4-methylpyridinium dibromide^43^ and 5-(4-(diphenylamino)phenyl)thiophene-2-carbaldehyde^44^ were synthesized according to a literature method.

^1^H spectra were measured on Bruker ARX 400 NMR spectrometers using CD_3_OD as the deuterated solvent. High-resolution mass spectra (HRMS) were recorded on a Finnegan MAT TSQ 7000 Mass Spectrometer system operating in the MALDI-TOF mode. UV absorption spectra were recorded on a Milton Ray Spectronic 3000 array spectrophotometer. Steady-state fluorescence spectra were recorded on a Perkin Elmer LS 55 spectrometer. Fluorescence images were collected on an Olympus BX 41 fluorescence microscope. Laser confocal scanning microscopy images were collected on a Zeiss laser scanning confocal microscope (LSM7 DUO) and analyzed using ZEN 2009 software (Carl Zeiss).

### Synthesis of TVP and TTVP

A solution of 4-(diphenylamino)benzaldehyde (54.6 mg, 0.2 mmol) and 1-(3-trimethylammoniopropyl)-4-methylpyridinium dibromide (71 mg, 0.2 mmol) was refluxed under nitrogen in dry ethanol catalyzed by a few drops of piperidine overnight. After cooling to room temperature, the solvent was removed by evaporation under reduced pressure. The residue was purified using a neutral aluminum oxide column using a DCM and methanol mixture (98 : 2 v/v) as the eluting solvent to give a red brown powder of TVP (92 mg, 66% of yield). ^1^H NMR (400 MHz, DMSO-D_6_), *δ* (ppm): 9.04 (d, *J* = 6.4 Hz, 2H), 8.23 (d, *J* = 6.8 Hz, 2H), 8.03 (d, *J* = 16.4 Hz, 1H), 7.64 (d, *J* = 8.8 Hz, 2H), 7.34–7.40 (m, 5H), 7.10–7.18 (m, 6H), 6.94 (d, *J* = 8.4 Hz, 2H), 4.61 (t, *J* = 7.2 Hz, 2H), 3.47 (t, *J* = 8.2 Hz, 2H), 3.12 (s, 9H), 2.46 (t, *J* = 7.4 Hz, 2H). ^13^C NMR (100 MHz, DMSO-D_6_), *δ* (ppm): 153.58, 149.54, 146.17, 144.22, 141.05, 129.87, 129.79, 127.97, 125.43, 124.55, 123.24, 120.64, 120.47, 61.77, 56.32, 52.42, 24.18. ESI HRMS: calcd for C_31_H_35_N_3_ [M – 2Br]^+^: 449.2831, found: 449.2823.

A solution of 5-(4-(diphenylamino)phenyl)thiophene-2-carbaldehyde (71 mg, 0.2 mmol) and 1-(3-trimethylammoniopropyl)-4-methylpyridinium dibromide (71 mg, 0.2 mmol) was refluxed under nitrogen in dry ethanol catalyzed by a few drops of piperidine overnight. After cooling to room temperature, the solvent was removed by evaporation under reduced pressure. The residue was purified using a neutral aluminum oxide column using a DCM and methanol mixture (98 : 2 v/v) as the eluting solvent to give a red brown powder of TTVP (98 mg, 71% of yield). ^1^H NMR (400 MHz, CD_3_OD), *δ* (ppm): 8.78 (d, *J* = 6.8 Hz, 2H), 8.13–8.17 (m, 3H), 7.58–7.60 (m, 2H), 7.48 (d, *J* = 4.0 Hz, 1H), 7.40 (d, *J* = 4.0 Hz, 1H), 7.30–7.34 (m, 4H), 7.02–7.12 (m, 9H), 4.60 (t, *J* = 7.8 Hz, 2H), 3.52–3.56 (m, 2H), 3.20 (s, 9H), 2.51–2.59 (m, 2H). ^13^C NMR (100 MHz, CD_3_OD), *δ* (ppm): 155.99, 150.81, 150.19, 148.72, 145.26, 140.33, 136.72, 135.83, 130.81, 128.12, 126.40, 125.16, 125.04, 124.90, 123.80, 121.71, 64.04, 58.04, 54.13, 26.27. ESI HRMS: calcd for C_35_H_37_N_3_S [M – 2Br]^+^: 531.2708, found: 531.2693.

### Cytotoxicity study

MTT assays were used to evaluate the cytotoxicity of the presented AIEgens. Cells were seeded in 96-well plates (Costar, IL, USA) at a density of 6000–8000 cells per well. After overnight culture, the medium in each well was replaced with 100 μL fresh medium containing different concentrations of TTVP. 24 hours later, 10 μL MTT solution (5 mg mL^–1^ in PBS) was added into each well. After 4 hours of incubation, 100 μL SDS–HCl aqueous solution (10% SDS and 0.01 M HCl) was added to each well. After incubation for 4 hours, the absorption of each well at 595 nm was recorded *via* a plate reader (Perkin-Elmer Victor3™). Each trial was performed with 6 wells in parallel.

### Cell imaging

Cells were grown in a 35 mm Petri dish with a coverslip at 37 °C. The live cells were incubated with a certain dye at a certain concentration for a certain time. After adding TTVP (500 nM), the Petri dish was shaken for a few seconds at room temperature, and then the coverslip was taken out. The TTVP-labelled cells were mounted and imaged using a laser scanning confocal microscope (LSM7 DUO) at 488 nm with 5% laser power (the scanning rate was 22.4 s per frame). The emission signal in the range of 600–744 nm was collected for cell imaging.

### Cytotoxicity to cancer cells under light irradiation

HeLa cells were seeded in 96-well plates (Costar, IL, USA) at a density of 6000–8000 cells per well. After overnight culturing, the medium in each well was replaced with 100 μL fresh medium containing different concentrations of TTVP. After incubation for 3 s, the plates containing HeLa cells were exposed to white light (around 10 mW cm^–2^) for 10 min, and another array of plates with cells were kept in the dark as the control. Then the plates were subjected to the same treatment as the biocompatibility test.

### Detection of cell necrosis/apoptosis induced by PDT

For the quantification of apoptosis and necrosis induced by PDT, cells were seeded in six-well plates at 2 × 10^5^ cells per mL, and allowed to grow for 24 h. TTVP aqueous solution was added to the cell well and incubated with cells for 20 min, and irradiated with white light at different time durations, and then another 24 h incubation was performed in the dark. After the treatment, the cells were collected and stained with Annexin V-FITC/PI. Each sample was analyzed using a flow cytometer (FACS Canto™ II, BD, USA).

### *In vivo* subcutaneous tumor imaging

All animal procedures were performed in accordance with the Guidelines for Care and Use of Laboratory Animals of South China Normal University and approved by the Animal Ethics Committee of South China Normal University. Female nude mice were purchased from the Animal Experiment Center of Southern Medical University. HeLa cells (1 × 10^6^) in PBS buffer were subcutaneously injected into the back of each female nude mouse (∼5 weeks old). After 7 days, the mice bearing HeLa tumors with an average volume of ∼60 mm^3^ were administered with TTVP at 10 μM by intratumor injection. The fluorescence imaging of tumor sites was performed from 10 min to 24 h post-injection. At 24 h post-injection, the mice were sacrificed, and major organs and tumors were collected, imaged, and analyzed by a histological assay. The excitation wavelength was set to be 543 nm.

## Conflicts of interest

There are no conflicts to declare.

## Supplementary Material

Supplementary information
