# Supplementary material for: Rational design of a water-soluble NIR AIEgen, and its application in ultrafast wash-free cellular imaging and photodynamic cancer cell ablation
Source: Chem Sci. 2018 Mar 13;9(15):3685–93. doi: 10.1039/c7sc04963c (PMC5935061; doi:10.1039/c7sc04963c)
Supplement: Supplementary file 1 [file SC-009-C7SC04963C-s001.pdf]

Electronic Supplementary Material (ESI) for Chemical Science.
This journal is © The Royal Society of Chemistry 2018

Copyright WILEY-VCH Verlag GmbH & Co. KGaA, 69469 Weinheim, Germany, 2016.

Supporting Information

Rational design of a water-soluble NIR AIEgen, and its applications for ultrafast wash-free cellular imaging and photodynamic cancer cell ablation

Dong Wang, Huifang Su, Ryan T. K. Kwok, Xianglong Hu, Hang Zou, Qianxin Luo, Michelle M. S. Lee, Wenhan Xu, Jacky W. Y. Lam, Ben Zhong Tang**

Table of Contents

Experimental procedures
Table S1. Optical properties of AIEgens TVP and TTVP.
Figure S1. ^{1}H NMR spectrum of TVP.
Figure S2. ^{13}C NMR spectrum of TVP.
Figure S3. HRMS spectrum of compound TVP.
Figure S4. ^{1}H NMR spectrum of 5-(4-(diphenylamino)phenyl)thiophene-2-carbaldehyde.
Figure S5. ^{1}H NMR spectrum of TTVP.
Figure S6. ^{13}C NMR spectrum of TTVP.
Figure S7. HRMS spectrum of compound TTVP.
Figure S8. Particle size distributions of TVP aggregates.
Figure S9. PL spectra of TTVP in solvents with different polarities.
Figure S10. Confocal images of HeLa cells stained with TTVP for different time.
Figure S11. Co-localization imaging of HeLa cells stained with TVP and DiO.
Figure S12. Plasma membrane-imaging of other cells.
Figure S13. Chemical trapping measurements of the $^{1}O_2$ quantum yield.
Figure S14. Decomposition rates of ABDA with light irradiation under different conditions.
Figure S15. Morphology change of plasma membrane upon light irradiation.
Figure S16. Co-localization imaging of COS-7 cells stained with TTVP and DiO.
Figure S17. PDT application of TTVP based on COS-7 cells.

Experimental procedures

Cell culture

HeLa cells were cultured in the MEM containing 10% FBS and antibiotics (100 units/mL penicillin and 100 mg/mL streptomycin) in a 5% CO_2 humidity incubator at 37 ^oC.

Confocal colocalization

After incubating HeLa cells with DiO at 37 ^oC for 10 min, TTVP was added into the culture, which was then shaken for a few seconds at room temperature. The medium was then removed and the cells were rinsed with PBS for three times and then imaged under confocal microscope. For TTVP, the emission filter was 600-744 nm; for DiO, the emission filter was 490–600 nm.

Photostability

The TTVP-labelled HeLa cells were imaged by a confocal microscope (Zeiss laser scanning confocal microscope LSM7 DUO) using ZEN 2009 software (Carl Zeiss). Conditions: for TTVP, excitation wavelength: 488 nm; for DiO, excitation wavelength: 488nm (5% laser power). 600–740 nm; for BODIPY493/503 Green, the excitation was 488 nm and the emission filter was 510–553 nm.

Singlet-triplet energy gap calculations

The calculation of excited state was performed at TD-DFT CAM-B3LYP/6-31G(d) level. The calculation of groud state and triplet state were performed at CAM-B3LYP/6-31G(d) level.

Table S1. Optical properties of AIEgens TVP and TTVP.

AIEgens	λ_{abs} [nm][a)]	ε (M^{-1} cm^{-1})	λ_{em} [nm] Soln (Φ_F)[b,c)]	Aggr(Φ_F)[c,d)]	Solid (Φ_F)[c,e)]	α_{AIE} ($I_{aggr,\,max}/I_{solu}$)	τ [ns][f)]
TVP	467	25070	619 (0.2%)	629 (4.4%)	657 (4.9%)	41.7	5.75
TTVP	480	33517	N/A	708 (1.7%)	705 (2.7%)	97.3	0.92

[a)]Absorption maximum in aqueous solutions; [b)]Emission maximum in aqueous solutions (10 μM); [c)]Fluorescence quantum yield determined by a calibrated integrating sphere; [d)]Emission maximum in aggregation state; [e)]Emission maximum in solid state; [f)]Fluorescence lifetime, measured under ambient conditions.

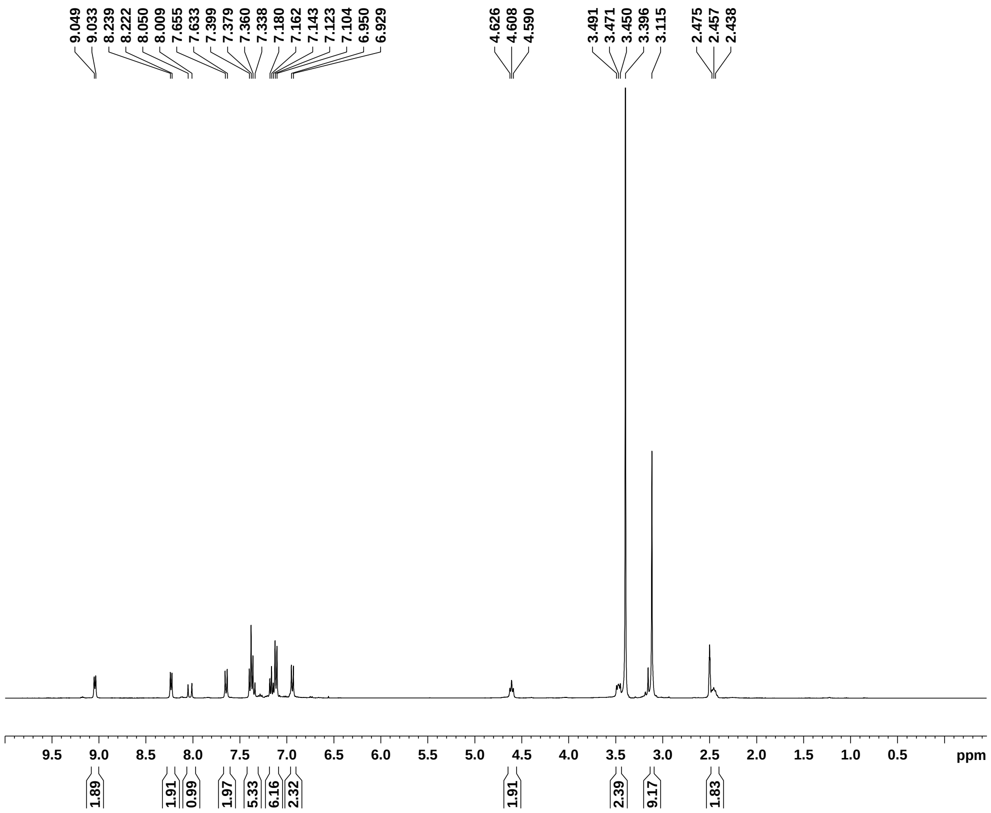

Figure S1. ^{1}H NMR spectrum of TVP.

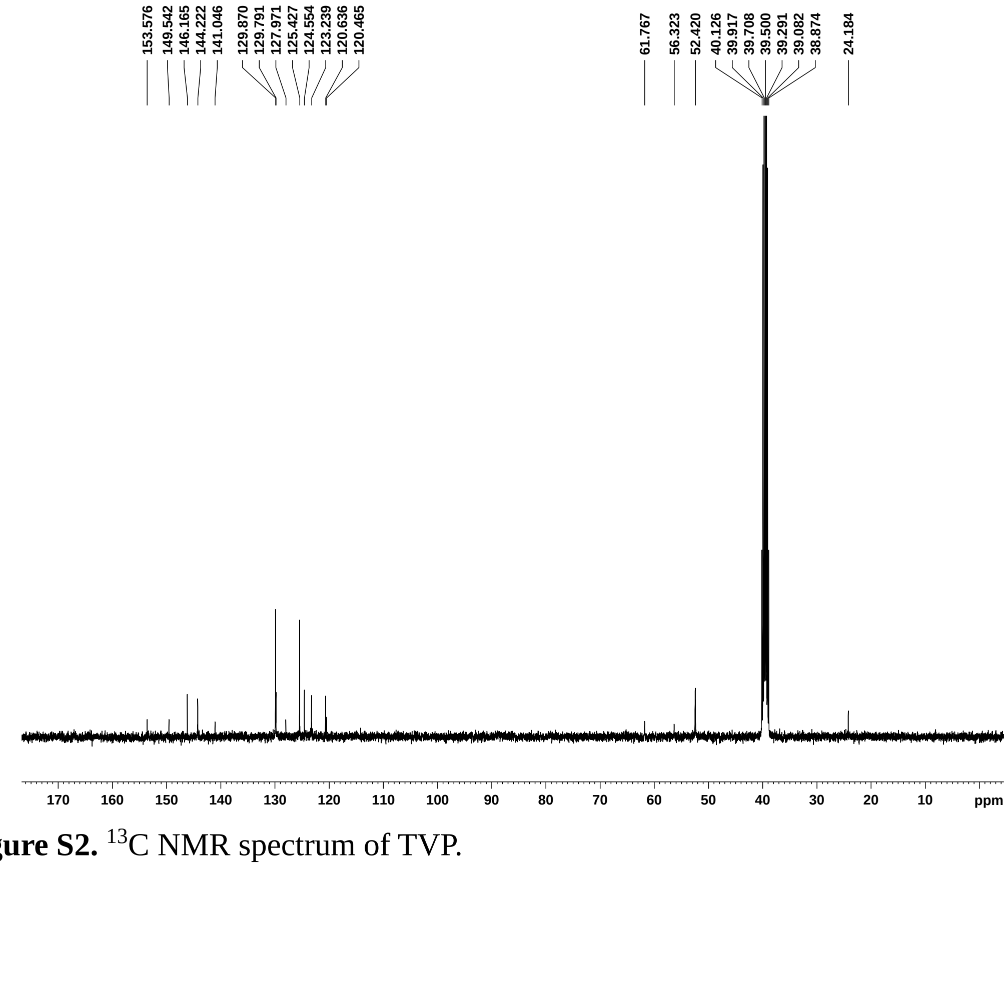

Figure S2. ^{13}C NMR spectrum of TVP.

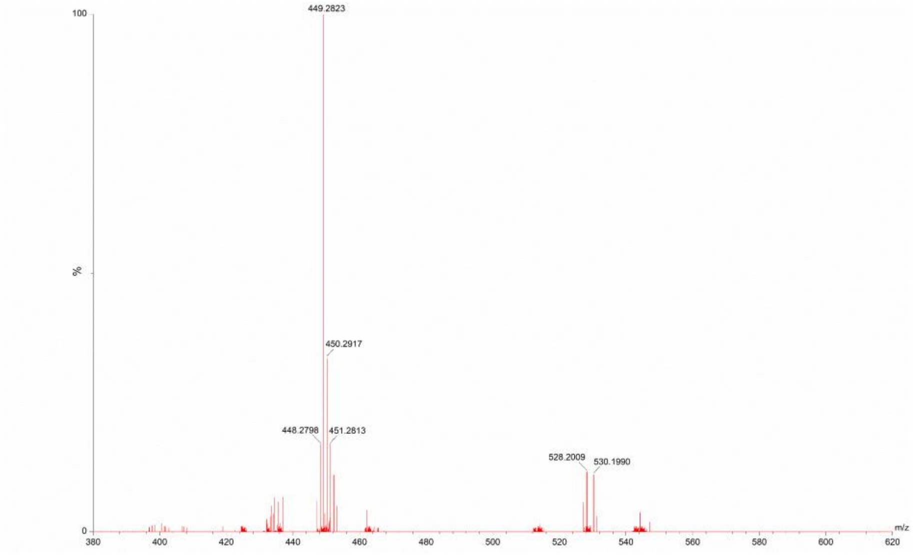

Figure S3. HRMS spectrum of compound TVP.

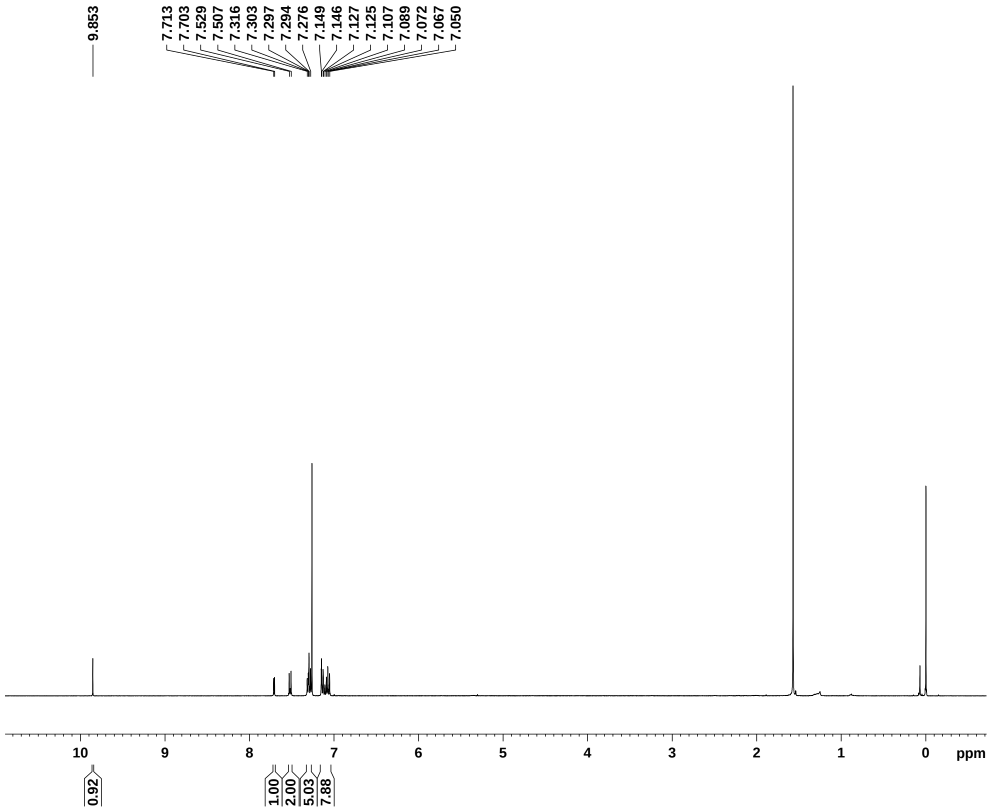

Figure S4. ^{1}H NMR spectrum of 5-(4-(diphenylamino)phenyl)thiophene-2-carbaldehyde.

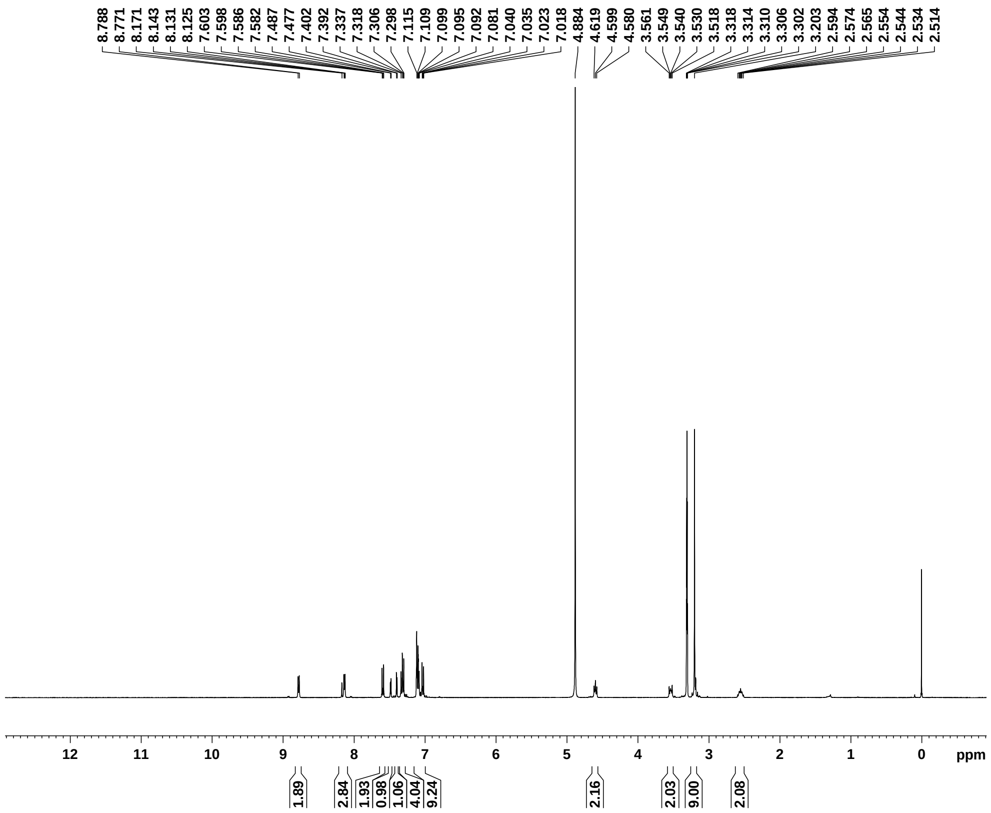

Figure S5. ^{1}H NMR spectrum of TTVP.

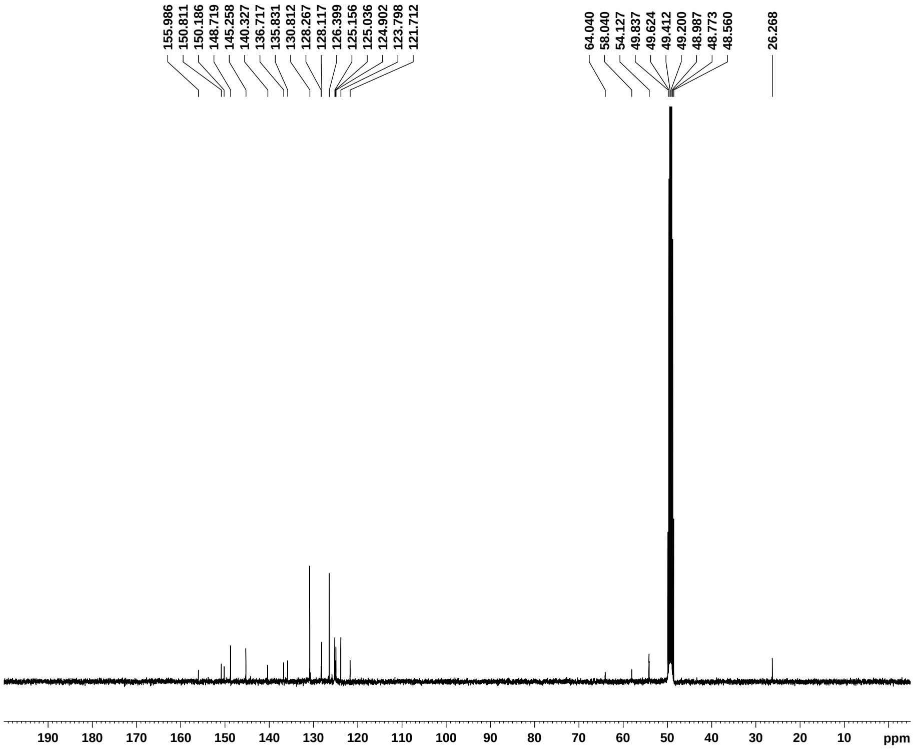

Figure S6. ^{13}C NMR spectrum of TTVP.

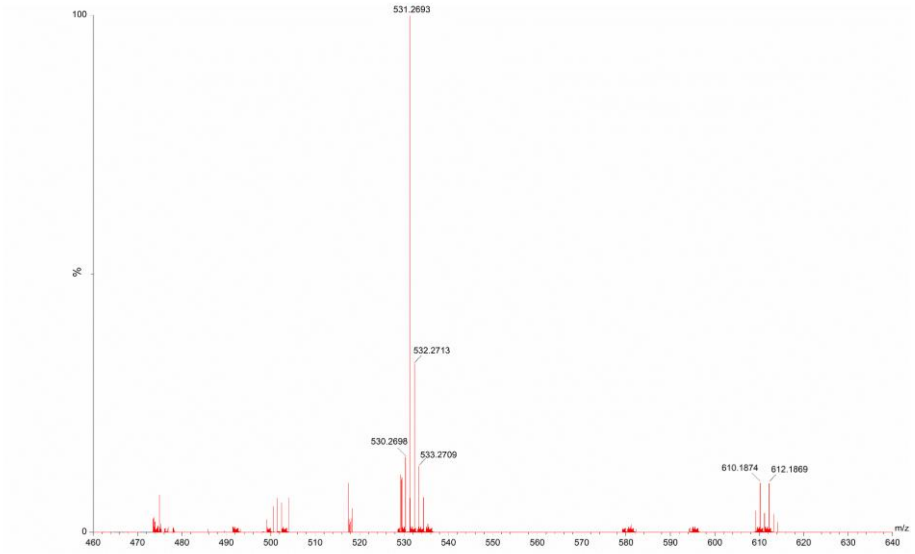

Figure S7. HRMS spectrum of compound TTVP.

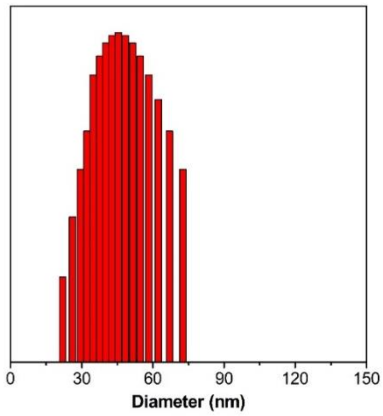

Figure S8. Particle size distributions of TVP aggregates in water/THF mixture with 90% THF fraction.

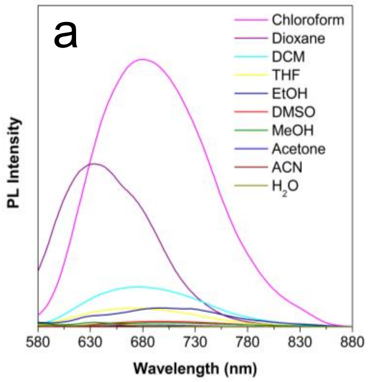

b

Solvent	λ_{em} (nm)
Chloroform	679
Dioxane	634
DCM	674
THF	676
EtOH	695
DMSO	696
MeOH	699
Acetone	n.d.
ACN	n.d.
H_2O	n.d.

Figure S9. a) PL spectra of TTVP in solvents with different polarities. Concentration: 0.5 μM; excitation wavelength: 480 nm. b) The data of emission maximum in (a).

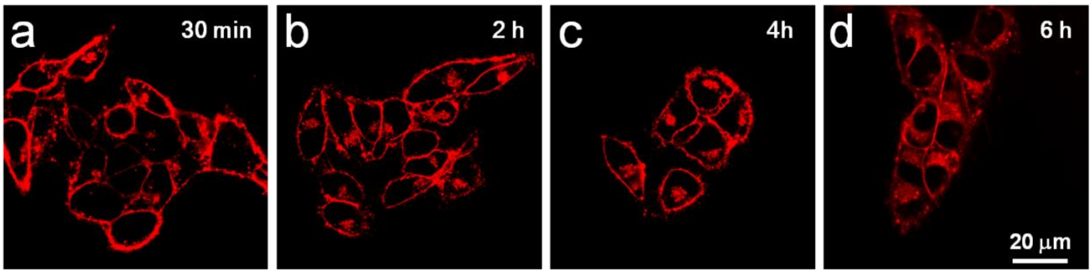

Figure S10. Confocal images of HeLa cells stained with TTVP for different time. Concentration: 500 nM. Scale bar = 20 μm. λ_{ex}: 488 nm (1% laser power, 0.05 μW).

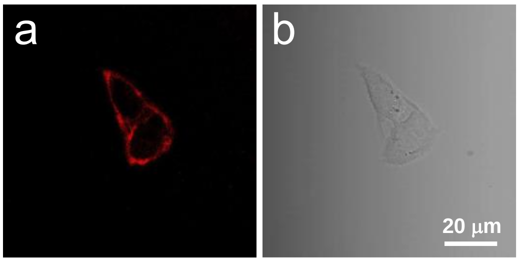

Figure S11. a) Confocal images and b) bright field of living HeLa cells after incubation with TVP (500 nM) by the use of washing procedure after incubation for 3 seconds. λex: 488 nm (1.2% laser power).

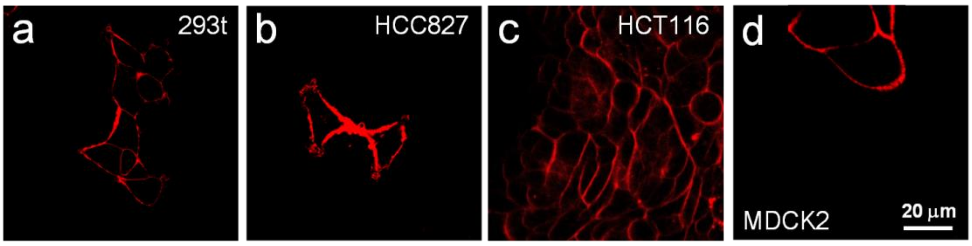

Figure S12. Extension of the wash-free and plasma membrane-specific imaging strategy to other cells. Confocal images of living a) 293T, b) HCC827, c) HCT116, and d) MDCK2 cells after incubation with TTVP (500 nM) for an extremely short incubation period (around 3 s). λ_{ex}: 488 nm (1% laser power, 0.05 μW).

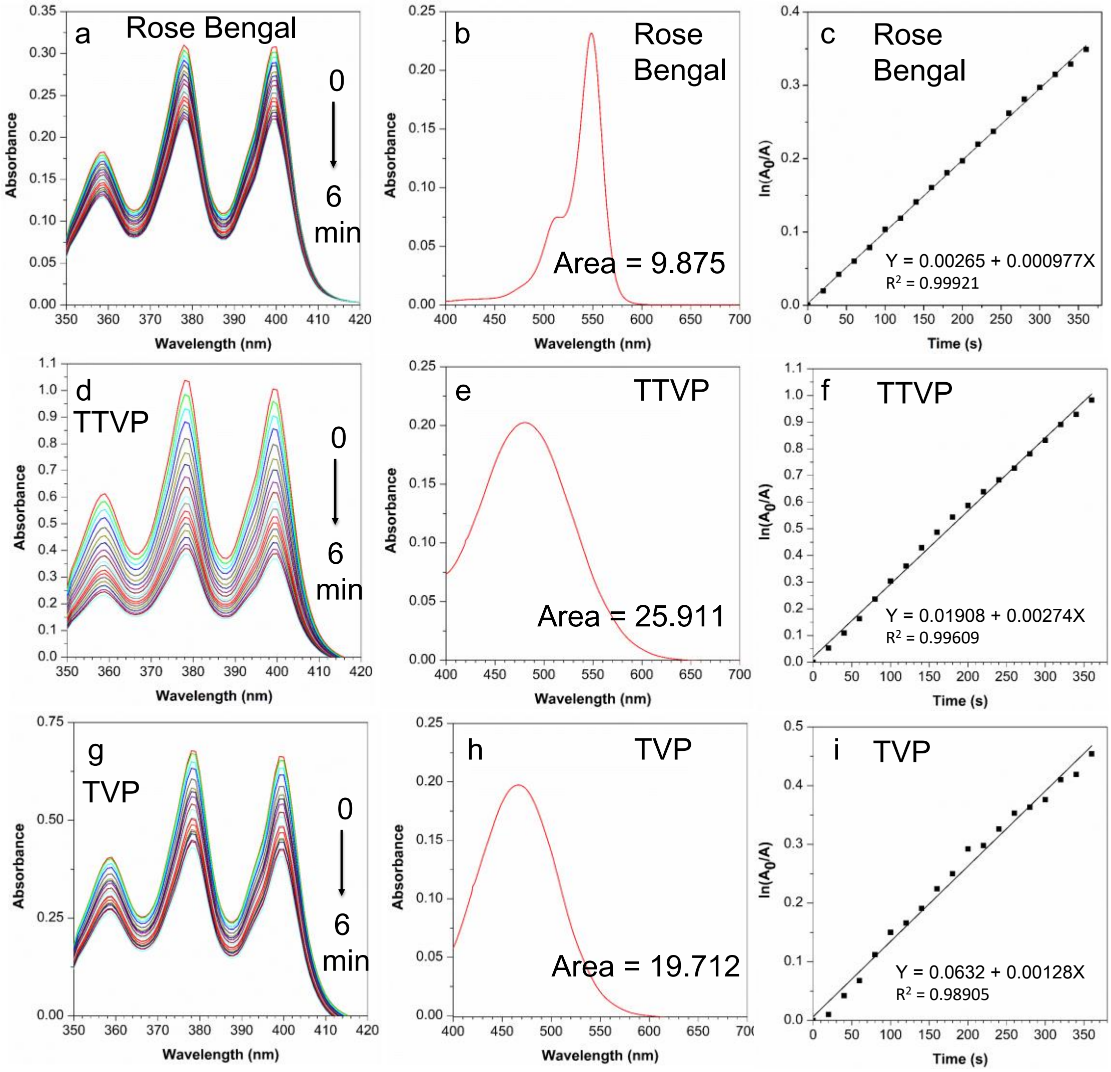

Figure S13. Chemical trapping measurements of the $^1O_2$ quantum yield. Photodegradation of ABDA with Rose Bengal (a), TTVP (d) and TVP (g). The absorption peak area of Rose Bengal (b), TTVP (e) and TVP (h). The decomposition rate constants of ABDA by Rose Bengal (c), TTVP (f) and TVP (I). To eliminate the inner-filter effect, the absorption maxima were adjusted to ~0.2 OD. These

measurements were carried out under white light irradiation in DMSO/water (v:v) = 1/100. [ABDA] = 10 × [AIEgens or Rose Bengal], time interval for recording the UV-vis spectra: 20 s.

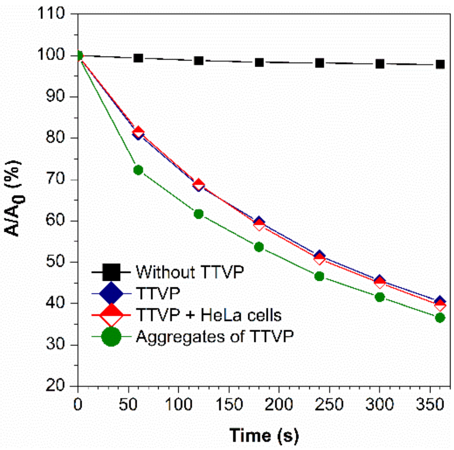

Figure S14. Decomposition rates of ABDA with light irradiation under different conditions, where A_0 and A are the absorbance of ABDA at 378 nm. [TTVP] = 5×10^{-6} M, [ABDA] = 5×10^{-5} M, time interval for recording the UV-vis spectra: 60 s.

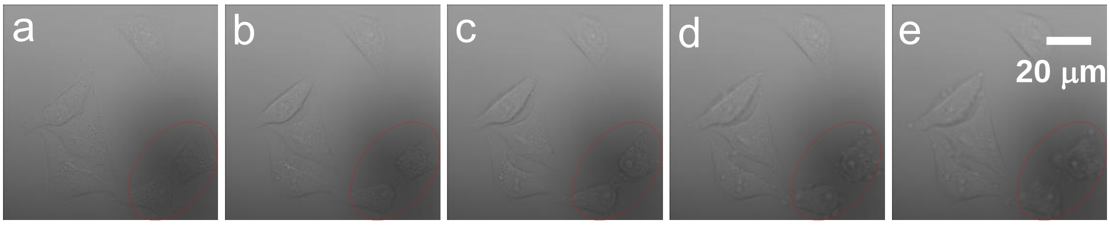

Figure S15. Bright-field of confocal images of HeLa cell stained with TTVP upon the increase of irradiation time. λ_{ex}: 488 nm (20% laser power, 0.925 μW).

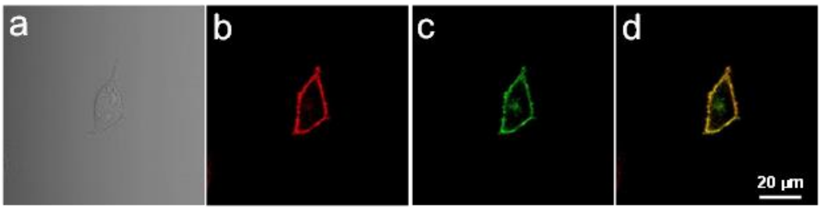

Figure S16. Co-localization imaging of COS-7 cells stained with TTVP and DiO. Confocal images of COS-7 cells stained with b) TTVP, c) CellMask Green, and d) merged images of panels b) and c), and a) Bright-field. Concentrations: TTVP (0.5 μM). The emission filter TTVP: 600-744 nm; the emission filter of CellMask Green: 490–565 nm.

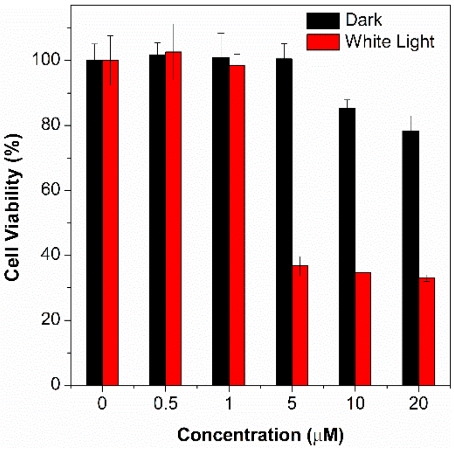

Figure S17. Cell viability of COS-7 cells stained with different concentrations of TTVP in the absence or presence of white light irradiation for 10 min. Light power: 10 mW/cm^2.
